# Ready to Go, But Not Alone: Why Post-Discharge Follow-Up Defines Hospital Length of Stay

**DOI:** 10.7759/cureus.86864

**Published:** 2025-06-27

**Authors:** George Bechir, Angelina Bechir

**Affiliations:** 1 Hospital Medicine, Franciscan Health, Munster, USA; 2 Pre-Medical Studies, Clemson University, South Carolina, Clemson, USA

**Keywords:** care transitions, culture result tracking, discharge planning, early discharge, follow-up systems, hospital readmission, length of stay, patient discharge confidence, post-discharge care, transitional care

## Abstract

A patient is told they are ready to go home, but no one calls about the biopsy, no results come in, and nothing happens. A culture result is finalized over the weekend, but there is no one assigned to follow up. The patient, once stable, is now back in the emergency room. These moments are not rare accidents; they are the predictable failures of a system that treats discharge as the end of care instead of the start of something crucial. This narrative review examines how hospitals can reduce the length of stay not by pushing patients out faster but by building systems that make earlier discharge feel safe for everyone involved. Drawing on 19 targeted studies and real-world protocols, we explore how structured post-discharge follow-up, including lab culture results (e.g., blood or urine) tracking, navigator roles, skilled nursing facility coordination, and patient communication tools, transforms discharge from a point of vulnerability into a confident transition. The data is clear: when patients know someone will follow through, and physicians trust that pending tasks will not fall through the cracks, discharge happens earlier, and readmission risk drops. Confidence, not just clinical stability, is the true threshold for going home. Hospitals that invest in continuity beyond their walls not just shorten stays but also change the story patients carry with them when they leave.

## Introduction and background

Every hospital struggles with the same tension: how do we safely move patients out of the hospital without making them feel abandoned? While clinical criteria might declare a patient "ready for discharge," patients themselves, and even their providers, may not feel ready. Behind every "medically cleared" order is a question: Who will follow up? What happens if results return abnormal? Will anyone call me? What if no one does? These are not trivial concerns. Fear of the unknown after discharge is one of the most common reasons patients linger in beds longer than necessary. From pending biopsy appointments to unresolved cultures or specialist decisions, patients often wait not for stabilization but for certainty. In a system increasingly focused on reducing length of stay (LOS) and improving throughput, these silent delays are costly and avoidable [1].

There is good news, however. Research shows that confidence, not just clinical metrics, may be the missing key to timely and safe discharge. Patients who feel supported, informed, and connected after discharge are more likely to leave on time, recover at home, and avoid unnecessary returns [2]. Similarly, when physicians trust that there is a reliable system for follow-up - when they know that pending test results such as cultures will be checked, specialists will be paged, and patients will have someone to call - they are more likely to feel comfortable discharging early [3].

Confidence-building systems are not just theoretical. Post-discharge navigators, follow-up hotlines, discharge coordinators, and structured communication channels have all been shown to reduce LOS while maintaining or improving patient satisfaction [4-6]. One study found that assigning a dedicated navigator to orthopedic surgery patients led to both shorter stays and higher satisfaction scores [4]. Another showed that structured discharge education with teach-back methods reduced readmissions [5]. Among patients with heart failure, higher levels of self-care confidence directly translated into better outcomes after going home [6]. Early physician follow-up for heart failure patients has also been shown to reduce 30-day readmissions, reinforcing the value of structured post-discharge care [7]. Effective post-discharge communication further supports patient recovery and continuity of care [8].

This is not just about saving hospital beds, although that matters, but also about changing the narrative. What if discharge was not the end of care but the beginning of a supported transition? What if patients left the hospital knowing someone was still watching out for them? And what if physicians discharged the patient not with worry but with trust in a system designed to catch what they might miss? This narrative review explores the practical, measurable, and patient-centered interventions that make this vision possible. Drawing on real-world examples and published evidence, we examine how structured post-discharge follow-up systems can reduce LOS, increase patient and provider confidence, and ensure that "safe to go home" truly means safe for everyone involved.

## Review

Methodology

This narrative review investigates the role of structured post-discharge follow-up systems in reducing hospital LOS, enhancing patient and provider confidence, and improving care transitions. The review focuses on interventions including transitional care strategies, culture and biopsy follow-up protocols, discharge navigation, and multidisciplinary planning models.

A comprehensive literature search was conducted to identify relevant English-language studies published between January 2013 and June 2025. PubMed served as the primary database for peer-reviewed studies, supplemented by Google Scholar to capture recent publications and gray literature, such as institutional reports and quality improvement initiatives. Search terms included combinations of "discharge planning", "hospital length of stay", "transitional care", "post-discharge follow-up", "culture result tracking", "readmission prevention", "patient navigation", and "provider confidence", connected by Boolean operators (AND, OR) to refine results. Additional sources were identified through manual citation tracking of key articles and review of hospital protocols from quality improvement reports.

Inclusion criteria prioritized peer-reviewed clinical studies, systematic reviews, and real-world implementation reports that addressed transitional care design and outcomes. Studies have shown that these failures are not simply administrative oversights; they represent a breakdown in transitional care. When patients do not receive timely communication about critical next steps, or when providers assume "someone else is handling it," the system fails [1-3]. This case underscores the importance of discharge as a coordinated handoff, not a cutoff. With structured post-discharge systems in place, such as a transition nurse to follow pending tests or a navigator to confirm biopsy scheduling, this patient might never have returned.

Why patient and provider confidence matters

Discharge is not merely a clinical endpoint; it is a psychological one. Patients do not just need to be stable; they need to feel safe going home. Likewise, providers do not just need to clear a patient medically; they need to trust that nothing critical will be missed after the patient leaves. In many hospitals, this dual confidence - patient and provider - is the true threshold for discharge. Patients who leave the hospital feeling unsure about what comes next are more likely to return unnecessarily.

In a large qualitative study, patients emphasized the emotional importance of continuity: knowing who to call, what warning signs to watch for, and how follow-up care would happen gave them peace of mind [2]. When those answers were unclear, patients expressed fear and even regret about having left too soon. On the provider side, hesitation is often driven not by the patient's current status, but by what is unresolved: pending cultures, unclear specialist plans, or anticipated delays in scheduling critical follow-up. A Cochrane review of discharge planning interventions noted that structured, individualized discharge plans not only reduced LOS but also increased provider satisfaction, largely due to improved confidence in continuity of care [3].

Confidence also has measurable effects. In a 2023 study on heart failure patients, those who scored higher in self-care confidence prior to discharge showed better adherence and outcomes after going home [6]. Similarly, Oh et al. demonstrated that structured discharge education reduced bounce-backs [5]. Discharge readiness, then, is not a fixed point on a clinical trajectory. It is a moment where medical stability intersects with emotional and logistical trust. When both the patient and provider feel confident, discharge becomes not a risk, but a relief.

Key pillars of a strong post-discharge system

Hospitals that discharge patients confidently and efficiently do not rely on good luck; they build systems. These systems are designed to anticipate the most common sources of post-discharge failure: missed results, unclear instructions, unreturned calls, and uncoordinated follow-ups. Successful programs share several foundational pillars that transform discharge from a point-in-time event into a reliable, supported handoff.

One such pillar is assigning a point person. Every patient discharged with a pending culture, imaging, or biopsy result should have someone specifically tasked with following that issue through to completion. Whether it is a discharge coordinator, transition nurse, or clinical pharmacist, the goal remains the same: ensuring continuity and accountability. In one large health system, assigning a discharge navigator to follow unresolved issues reduced readmissions and improved both staff and patient satisfaction [4].

Another critical pillar is tracking pending results daily. Pending cultures, pathology, or imaging studies should never fall into a black hole. A simple, shared tracking list that is monitored daily by a designated team member ensures that follow-up actions are not missed. Structured follow-up systems have been shown to enhance patient engagement and reduce readmission risks [9]. Gallagher et al. demonstrated that pharmacist-led discharge medication reconciliation significantly reduced unplanned hospital readmissions [10].

A third essential practice is initiating post-discharge communication planning at the time of admission. This approach is key to reducing hospital LOS. Long’s health C3 model illustrates that establishing structured follow-up pathways early, such as assigning coordinators to arrange patient hotlines or schedule follow-up calls within the first 24 hours, builds early confidence in care transitions [11]. By proactively implementing these systems, hospitals address many patient and provider concerns before they arise, allowing for earlier and safer discharges without compromising quality or continuity of care.

Reducing length of stay for patients with pending cultures or biopsy results

One of the most common and solvable causes of prolonged hospitalization is the presence of pending test results, such as microbiology cultures or biopsy reports, at the time a patient is otherwise medically stable. These patients often do not require acute inpatient care, yet they remain in beds because physicians are hesitant to discharge them without clear follow-up systems in place. This hesitancy is understandable, as a positive culture or a missed result can carry serious consequences if not acted upon promptly. However, holding patients in the hospital "just in case" is rarely the best solution, especially when a structured follow-up process can ensure that those pending results are tracked, acted upon, and communicated safely after discharge.

Patient surveys have shown that confidence in post-discharge support is a key determinant of whether patients feel ready to go home [12]. Structured communication channels, such as dedicated contact points, further enhance this confidence [5]. Providers, too, are more willing to discharge when they trust that culture results or pathology updates will not be missed [13]. The U.S. Agency for Healthcare Research and Quality (AHRQ)’s IDEAL discharge planning framework explicitly recommends that all pending results be clearly documented, tracked, and explained to patients before discharge to avoid gaps in care [14]. Implementing a post-discharge culture tracking system, where a pharmacist or discharge coordinator monitors outstanding results daily and contacts infectious disease or the primary team if escalation is needed, has been shown to reduce readmissions and build provider confidence in early discharge [10].

When combined with reliable contact numbers for patients and a clear protocol for skilled nursing facility or outpatient communication, this approach allows safe discharge even before results are finalized. Multidisciplinary involvement is key. Nurses, case managers, infectious disease consultants, and skilled nursing facilities must all be aligned on how to manage late-arriving results. Physiopedia’s discharge planning model emphasizes the need for shared accountability across the care team to ensure smooth transitions [15]. Ultimately, patients with pending cultures or biopsy results do not need prolonged admission; they need a system that ensures follow-up happens, even after they go home.

Post-discharge tracking systems

Discharging a patient before all cultures or biopsies have been finalized requires more than confidence. It requires a system. Without reliable post-discharge tracking, the responsibility to follow up often falls unevenly or is lost entirely. That is when missed results, failed handoffs, and unnecessary readmissions occur. However, when hospitals implement structured follow-up protocols, both safety and provider confidence increase, making earlier discharge decisions easier [6].

A simple but effective tool is a daily tracking list of patients discharged with pending microbiology cultures, imaging studies, or pathology reports. This list should be managed by a designated team member, often a discharge coordinator, clinical pharmacist, or nurse navigator, who checks daily for finalized results, flags abnormal findings, and communicates necessary follow-up actions to the appropriate clinician or care team.

This approach was central to the Connect Care EDD system, where patients discharged with outstanding issues were tracked using a standardized daily report. The tracking staff alerted teams when cultures returned, helped adjust antibiotics, and coordinated with outpatient clinics or skilled nursing facilities to communicate changes in care plans. This intervention was linked to improved care continuity and reduced LOS [11].

Hospitals that embed follow-up into their operational culture are the ones best positioned to reduce LOS safely. General follow-up care, including communication and engagement strategies, supports safe transitions and reduces readmissions [8,9]. Bravo’s work on early discharge and other initiatives with dashboards and checklists improved satisfaction and reduced readmissions [16]. Importantly, these systems were not complex. Many were built in Excel or layered onto existing EMR tools. What mattered was that someone was responsible, every day, for closing the loop.

Effective tracking systems work best when embedded in multidisciplinary discharge planning. For patients discharged to skilled nursing, close communication with those facilities is essential. They must be able to receive updated results, modify antibiotics if necessary, and respond quickly to changes in care plans. Tracking systems help coordinate those efforts and prevent post-discharge fragmentation. Ultimately, the question is not whether test results will come back after discharge. It is whether your hospital will know and respond when they do.

Policy and institutional considerations

For post-discharge follow-up systems to be effective, they must be supported not only by individuals but also by institutional policy. Without standardized expectations and structured workflows, even the most well-intentioned discharge plans can fail at the point of execution. Institutions must ensure that all involved teams understand their roles and responsibilities in the discharge process.

A key area for improvement is the coordination with skilled nursing facilities. Berkowitz et al. showed that a structured discharge process in these settings reduced hospital readmissions and eased inpatient bottlenecks [17]. Institutions must verify that skilled nursing facilities are equipped to accept patients with pending cultures, titrate antibiotics after discharge, and perform procedures such as inserting peripherally inserted central catheters when needed. Without this readiness, patients may remain hospitalized longer than necessary for services that could otherwise be delivered post-discharge.

Another important area of focus is the issue of weekend discharges. These are often avoided due to limited access to infectious disease consults or unclear pathways for managing changes in patient care after discharge. Many providers hesitate to send patients home on a Friday if pending results or unresolved questions remain. However, this hesitation can be addressed with preplanned weekend discharge protocols. Hospitals that implemented structured discharge planning for weekends reported lower readmission rates and more timely discharges [18].

Accountability after discharge must also be clearly defined. There should be no ambiguity regarding who is responsible for reviewing pending test results and following up with the patient, skilled nursing facility, or pharmacy. Brunner-La Rocca et al. emphasized that chronic disease management gaps significantly contribute to hospital readmissions, highlighting the importance of post-discharge accountability [19]. These responsibilities should be incorporated into the electronic medical record workflows, supported by ongoing staff training, and discussed routinely during discharge planning rounds. Clear documentation and consistent use of teach-back methods have been shown to improve patient understanding and readiness for discharge [5].

Ultimately, when institutions develop policies that establish clear roles, improve communication across settings, and support safe transitions, discharge becomes a coordinated and reliable process. Hospitals do not need to rely on assumptions or good intentions. Proven policies and systems already exist that reduce LOS while maintaining high standards of care. The challenge lies in making these practices standard and sustainable across all care settings.

Patient communication and follow-up support

Discharge plans must include a clear communication pathway for patients. Every patient should leave the hospital with a reliable phone number, such as a nurse line, discharge navigator, or designated care liaison, to call in case of questions or concerns. This support helps reduce anxiety and reinforces continuity of care. While it may not be feasible for every physician to call each patient, a structured system should ensure follow-up contact within a reasonable time frame [7]. Ideally, a care team member, such as a transition nurse or discharge coordinator, reaches out to confirm that instructions are understood, symptoms are stable, and no barriers exist to safe recovery.

In addition, hospitals should implement predefined coordination pathways with key specialties to ensure continuity and confidence in post-discharge care. For example, cardiology assessments such as stress tests or angiograms should be scheduled in the near future for patients with musculoskeletal-appearing chest pain to ensure timely evaluation [20]. Patients with gastrointestinal bleeding that appears to be due to hemorrhoids should receive gastroenterology follow-up with outpatient endoscopy arranged before discharge [21]. When procedures are required but not urgent, interventional radiology can schedule outpatient or skilled nursing facility-based care to avoid unnecessary inpatient stays. Furthermore, arranging visits with primary care providers or specialty consultants shortly after discharge, ideally within a few days, has been shown to significantly reduce readmission risk in heart failure and general medical populations [22,23]. These steps not only improve the transition from inpatient to outpatient care but also provide assurance to both patients and providers that the next steps are clear, timely, and coordinated.

Embedding these coordination efforts into discharge planning builds both patient and provider confidence, supports safer early discharge, and strengthens continuity of care beyond the hospital walls.

## Conclusions

The decision to discharge a patient is rarely just clinical - it is emotional, logistical, and deeply human. Even when vital signs are stable and treatment is complete, discharge may feel premature if patients do not understand what happens next or if physicians worry that follow-up systems will fail. These moments of doubt often translate into longer stays, higher costs, and avoidable readmissions. But it does not have to be this way. When hospitals build structured post-discharge follow-up systems - assigning clear responsibility for pending results and creating communication pathways and coordination with a care structure team to discharge patients safely - they remove the ambiguity that drives unnecessary inpatient care days.

These systems do more than move patients; they build trust - trust that someone will call, that results will be checked, that the care does not stop at the hospital door. Patients who feel supported are more willing to leave. Providers who trust the system are more willing to discharge. Studies consistently show that follow-up mechanisms increase both confidence and safety across multiple clinical settings, ranging from surgery to stroke to heart failure. If hospitals want to reduce LOS while preserving quality, they must start with the end in mind. Early discharge is not a risk when it is backed by a system designed to support and catch the loose ends. It is a partnership between care teams, patients, and a structure that says: “We’ve got you,” even after you leave.
